# Enhancement of Zn tolerance and accumulation in plants mediated by the expression of *Saccharomyces cerevisiae* vacuolar transporter *ZRC1*

**DOI:** 10.1007/s00425-021-03634-z

**Published:** 2021-05-06

**Authors:** Giovanni DalCorso, Flavio Martini, Elisa Fasani, Anna Manara, Giovanna Visioli, Antonella Furini

**Affiliations:** 1 Department of Biotechnology, University of Verona, Strada Le Grazie 15, 37134 Verona, Italy; 2 Department of Chemistry, Life Sciences and Environmental Sustainability, University of Parma, Parma, Italy

**Keywords:** Heavy metals, Metal accumulation in plants, Phytoremediation, *Populus alba*, ZRC1 transporter

## Abstract

**Main conclusion:**

Transgenic *Arabidopsis thaliana* and *Populus alba* plants overexpressing the zinc transporter *ScZRC1* in shoots exhibit Zn tolerance. Increased Zn concentrations were observed in shoots of *P. alba*, a species suitable for phytoremediation.

**Abstract:**

Genetic engineering of plants for phytoremediation is worth to consider if genes leading to heavy metal accumulation and tolerance are expressed in high biomass producing plants. The *Saccharomyces cerevisiae ZRC1* gene encodes a zinc transporter which is primarily involved in the uptake of Zn into the vacuole. The *ZRC1* gene was expressed in the model species *A. thaliana* and *P. alba* (cv. Villafranca). Both species were transformed with constructs carrying *ScZRC1* under the control of either the CaMV35S promoter for constitutive expression or the active promoter region of the tobacco Rubisco small subunit (pRbcS) to limit the expression to the above-ground tissues. In hydroponic cultures, *A. thaliana* and poplar *ScZRC1*-expressing plants accumulated more Zn in vegetative tissues and were more tolerant than untransformed plants. No differences were found between plants carrying the CaMV35::*ScZRC1* or pRbcS::*ScZRC1* constructs. The higher Zn accumulation in transgenic plants was accompanied by an increased superoxide dismutase (SOD) activity, indicating the activation of defense mechanisms to prevent cellular damage. In the presence of cadmium in addition to Zn, plants did not show symptoms of metal toxicity, neither in hydroponic cultures nor in soil. Zn accumulation increased in shoots, while no differences were observed for Cd accumulation, in comparison to control plants. These data suggest that ectopic expression of *ScZRC1* can increase the potential of poplar for the remediation of Zn-polluted soils, although further tests are required to assay its application in remediating multimetal polluted soils.

**Supplementary Information:**

The online version contains supplementary material available at 10.1007/s00425-021-03634-z.

## Introduction

Environmental heavy metal pollution is a worldwide issue that arises mainly from industrial activities, mining and agricultural practices (Nriagu and Pacyna 1988). The heavy metal pollution can cause a significant decrease in plant yield and can be dangerous for all living organisms. Plants are likely the entry point of heavy metals into the food chain, with subsequent concerns for animal and human health (Peralta-Videa et al. 2009). Environmental research aims to both, avoid pollution and remediate already contaminated lands, with the least possible impact on the ecosystem. However, biological remediation of metal-polluted soils can prove challenging, since contrary to organic compounds, biodegradation of heavy metals is impossible and hence they continuously accumulate in the environment (Shim et al. 2013; He et al. 2015a, b). To this respect, plants can be exploited to help detoxifying polluted soils, due to their ability in the uptake and root-to-shoot transport of heavy metals. This approach is known as phytoremediation (Salt et al. 1998; McGrath and Zhao 2003). It has attracted attention during the last decades as a non-invasive and cost-effective strategy, alternative or complementary to currently adopted chemical and physical approaches (Pilon-Smits 2005; Zhao et al. 2009). Phytoremediation encompasses two main processes: (i) pollutant containment and stabilization, when the vegetation covers the polluted areas, preventing migration to ground waters and nearby areas, thus reducing the inlet of toxic metals into the food chain (Raskin and Ensley 2000) and (ii) extraction in which the plant species accumulate pollutants in harvestable tissues. The latter has the advantage that the heavy metal is permanently removed, and in some cases, can be recovered from the plant tissues (Elekes 2014). Ideally, at the end of the process the concentration of heavy metals in the shoot biomass should be higher than that present in the soil.

Zinc (Zn) is an essential nutrient for all organisms and is implicated in many biological processes. Among prokarya and eukarya, between 4 and 10% of the genomes encodes Zn proteins (Andreini et al. 2006). This metal is required in the catalytic processes of several metalloenzymes and is a highly effective structural cofactor of many proteins (Krämer and Clemens 2005; Maret 2009). However, despite its essential role, Zn can be toxic when present in excess. Therefore, the intracellular Zn concentration has to be tightly controlled to avoid toxicity and competition with other metal cations that would result in impaired uptake and inhibition of the function of non-Zn proteins by occupying binding sites (Sinclair and Krämer 2012). Zn is usually abundant in the mineral component of soils. Its levels have increased through human activities such as mining and smelting, and agricultural practices such as the intensive use of phosphate-based fertilizers or the application of sewage sludge (Chaney 1993). Organisms have evolved mechanisms of Zn homeostasis to ensure, to some extent, adequate intracellular levels in response to environmental changes in Zn content; however, Zn excess can prove very detrimental for their metabolism and physiology. In plants, toxicity symptoms include reduced yield and stunted growth, although crops differ markedly in their susceptibility to Zn toxicity (Broadley et al. 2007). To approach this problem, the application of phytoremediation to Zn-polluted soils can prove beneficial. Metal hyperaccumulators such as *Arabidopsis halleri* and *Noccaea caerulescens* have the capacity to remove Zn from soil, but their small biomass makes them unsuitable for phytoremediation purposes. The ideal species should grow fast, have a deep and extensive root apparatus along with a consistent development of biomass and, not least, it must tolerate heavy metal pollution (Halimaa et al. 2014).

Poplars (*Populus* spp.) are fast growing trees, characterized by deep root system and large aerial biomass, which can be obtained within few growing seasons in the temperate zone (Yadav et al. 2010; Bredemeier et al. 2015). They are easily propagated, have the aptitude to be coppiced (Rockwood et al. 2004), and have well-established industrial uses such as bioenergy, pulp and paper production, as well as ecological applications in the form of windbreaks and shelterbelts (da Ros and Mansfields 2020). Most importantly, as poplars are not a source of food, the risk of heavy metals entering the food chain is low. *Populus* spp. have been tested for their heavy metal tolerance and accumulation. It was demonstrated that intra-population genetic variability could affect heavy metal accumulation: clones tolerant to copper (Cu) and Zn showed very different content of Cd, lead (Pb), Zn and iron (Fe), and different ability of metal translocation (Baldantoni et al. 2014). In trials applying nutrient films with a mix of trace elements, a screen of 21 clones of poplar revealed variability between clones in element accumulation and underlined the effect of pH and multi-elemental pollution on the growth of the above-ground biomass (Migeon et al. 2012). Cu, Cd, Pb and Zn remediation by *Populus* inoculated with arbuscular mycorrhizae was also demonstrated (Bissonnette et al. 2010). Many other works have compared the response of poplar clones in hydroponic medium for selecting the best genotype for phytoremediation (Vassilev et al. 2005; Dos Santos Utmazian et al. 2007; Zacchini et al. 2009). In addition, the genome sequencing of *Populus trichocarpa* (Tuskan et al. 2006), and recently whole-genome resequencing of more than 500 individuals of the same species, revealed a high degree of adaptive traits across a wide range of latitude (Evans et al. 2014). All these features make *Populus* spp. an appropriate genus to be considered in phytoremediation approaches. Moreover, the established genetic transformation protocols (Balestrazzi et al. 2000) allow to improve useful traits such as heavy metal tolerance and accumulation. For instance, the transformation of yellow poplar (*Liriodendron tulipifera*) with the bacterial *merA* gene provided a system for mercury (Hg) remediation of polluted sites by the conversion of highly toxic Hg(II) to less toxic elemental Hg(0) (Rugh et al. 1998). Transgenic poplar lines were also designed for the remediation of organic xenobiotics, by overexpressing gamma-glutamylcysteine synthetase for treating chloroacetanilide herbicides (Gullner et al. 2001). More recently, poplar was engineered to improve Cd phytoremediation by introducing the yeast vacuolar Cd-transporter ScYCF1. Plants overexpressing *ScYCF1* displayed greater tolerance to Cd and their shoots contained five times more Cd than wild-type plants (Shim et al. 2013). Usually, the excess of heavy metals, both nutrients and toxic ions, is stored in the vacuole (Martinoia et al. 2012), making this compartment a good target to deliver potentially toxic heavy metals. Accumulating metals into the vacuole could increase tolerance and, indirectly, phytoremediation potential (Tong et al. 2004).

The objective of this work was to engineer *Arabidopsis thaliana*, as model species, and white poplar (*Populus alba* L. clone Villafranca) with the introduction of the *Saccharomyces cerevisiae ZRC1* gene to increase the Zn accumulation potential. This poplar clone, obtained at the Poplar Research Institute, Casale Monferrato (Italy), was registered for commercial use in Italy in 1989; it is used for reforestation in the plains along rivers, showing good biomass production and remarkable resprouting ability after coppicing (Confalonieri et al. 2000). In addition to these features, this clone was selected as the most tolerant and best accumulating in a Zn dose–response study with four commercial clones (Romeo et al. 2014). The *ScZRC1* gene encodes a high-affinity vacuolar Zn transporter belonging to the CDF (Cation Diffusion Facilitator) transporter family, primarily involved in the uptake of Zn into the vacuole (MacDiarmid et al. 2003), and, to a lesser extent, Cd and nickel (Ni) (MacDiarmid et al. 2002). The transgenic lines were designed to express *ScZRC1* under the control of the CaMV35S constitutive promoter, to have protein expression and hence metal accumulation in the whole plant, or driven by a light-inducible promoter (pRbcS) to direct metal accumulation mainly to the aerial tissues. Results of this work demonstrated that the engineered plants perform better in term of stress tolerance and biomass production even in control conditions and are efficient in the accumulation of Zn, shedding lights on their possible consideration into phytoremediation application.

## Materials and methods

### Plant materials, metal treatment and growth conditions

White poplar (*Populus alba* L. clone Villafranca), kindly provided by Dr. G. Nervo (Research Unit for Intensive Wood Production, Casale Monferrato, Alessandria, Italy), was propagated in vitro. Plants of *A. thaliana* (Col 0) were cultured on a MS (Murashige and Skoog 1962) medium, whereas poplar cuttings were maintained on a WPM medium (Lloyd and McCown 1981) in a growth chamber under 16-h light/8-h dark regime at 22 °C/18 °C (light intensity of 80 to 120 μmol m^−2^ s^−1^). For in vitro culturing, seeds of wild type and transformed *A. thaliana* were surface-sterilized with 70% ethanol for 1 min, and then with 10% sodium hypochlorite containing 0.03% TritonX-100 for 15 min, before being rinsed three times with sterile water. Sterile seeds were sown on solid MS medium supplemented with 30 g L^−1^ sucrose (with the addition of 100 mg L^−1^ kanamycin for the selection of transformed lines) and vernalized for 2 days at 4 °C prior to be transferred to the growth chamber. Alternatively, plants were grown in hydroponic culture in Hoagland’s solution (Hoagland and Arnon 1950) in the growth chamber, under controlled conditions. Two-week-old *Arabidopsis* plants were submitted to the following treatments: (i) control conditions in standard Hoagland’s solution (0.7 µM Zn) and (ii) with the addition of 20 µM ZnSO_4_. This Zn concentration was chosen as high but below toxicity levels and, therefore, tolerated by the non-tolerant species *A. thaliana* (Fasani et al. 2017). Two-week-old (roughly 10 cm tall) in vitro propagated poplar plants were transferred for 2 weeks in Hoagland’s solution, and thereafter submitted to metal treatment with 500 µM ZnSO_4_ (Romeo et al. 2014) or 10 μM CdSO_4_ and 250 μM ZnSO_4_ for further 3 weeks. In this case, plant conditions before treatment were adopted as control condition. For the in soil experiment on poplar, plastic pots were filled with 750 g of a mixture (1:1) peat: sand and saturated with Hoagland’s solution (avoiding leakage) with additional CdSO_4_ and ZnSO_4_. The obtained artificially polluted soil was characterized by pH 6.7 and a metal contamination corresponding to 10 mg kg^−1^ CdSO_4_ and 300 mg kg^−1^ ZnSO_4_, consistently with the concentration reported in literature for artificially contaminated soils (Guerra et al. 2011). Two-week-old in vitro propagated poplar plants were transferred into normal non-contaminated soil and acclimated for 2 weeks. Afterwards, plants were transferred to the above-described contaminated pots and grown for further 3 weeks, watered every week with an equal amount of water, avoiding leakage from the pots.

### Plasmid DNA constructs and plant transformation

The *ScZRC1* full-length coding sequence was amplified from the genomic DNA of *S. cerevisiae*; the gene-specific primers for the amplification include the restriction sites for the subsequent cloning into the destination vector, *Xba*I and *Sac*I, at the 5′-end (forward 5′-TCTAGAATGATCACCGGTAAAGAATTGA-3′ and reverse 5′-CTCGAGTTACAGGCAATTGGAAGTATTG-3′). The amplified fragment was subjected to digestion with *Xba*I and *Sac*I and subsequent ligation into the plant expression vector pMD1 (Sendín et al. 2012), under the control of the CaMV35S promoter. In addition to the constitutive promoter, a construct was produced to limit the expression to the above-ground tissues. In this case, the CaMV35S promoter in the above-mentioned construct was replaced with the 1050 bp-long regulatory region upstream the Rubisco small subunit (RbcS) gene. To obtain this, the Rbcs promoter was amplified from tobacco genomic DNA (*Nicotiana tabacum* cv. Petit Havana SR1, Uozumi et al. 1994; Cui et al. 2015) by means of specific primers which include the restriction sites *Hind*III and *Xba*I at the 5′-end (forward 5′-AAGCTTAAGCTTGTGGGAACGAGATAA-3′ and reverse 5′-TCTAGATGTTAATTACACTTAGACAGAAAG-3′). The amplified fragment was cloned into the pMD1 vector containing the ScZRC1 sequence, by *Hind*III and *Xba*I restriction (the two restriction sites flank the CaMV35S sequence in the pMD1 vector) and subsequent ligation, replacing the CaMV35S sequence. The recombinant plasmids were checked by sequencing, transformed into *Agrobacterium tumefaciens* strain EHA105 and used to transform *A. thaliana* and white poplar. The former was transformed using the floral dip method (Zhang et al. 2006), whereas poplar transformation was accomplished by co-cultivation for 8–10 min of an *A. tumefaciens* solution with leaf discs of in vitro plantlets, followed by regeneration as previously described (Fan et al. 2015). Transformation was confirmed in both species by PCR analysis on genomic DNA and evaluation of transgene expression. In all subsequent experiments, wild-type plants were adopted as control in both poplar and *A. thaliana*.

### Genomic DNA isolation, RNA extraction and cDNA synthesis

Genomic DNA was extracted from plant tissues using the Qiagen Genomic DNA Extraction Kit (Qiagen, Hilden, Germany). Total RNA was extracted from fresh tissues with TRIzol Reagent (Thermo Fisher Scientific, Waltham, MA, USA). After DNase treatment, first-strand cDNA was synthesized using SuperScript III Reverse Transcriptase kit (Thermo Fisher Scientific) before real-time reverse transcription polymerase chain reaction (real-time RT-PCR) experiments.

### Expression analysis in transgenic *A. thaliana* and poplar plants

Expression of target genes in the *Arabidopsis* and poplar transgenic lines was assessed by real-time RT-PCR performed with a StepOne Plus Real-Time PCR System (Applied Biosystems) using KAPA SYBR FAST ABI Prism 2X qPCR Master Mix (Kapa Biosystems, Wilmington, MA, USA). Each reaction (40 amplification cycles) was carried out in triplicate and a melting curve analysis was used to confirm the amplification of specific targets. Specific primers were used to specifically amplify *ScZRC1* (forward 5′-AGCTCCGCCAAGCTGATAAG-3′ and reverse 5′-TCTGCGAATATCCTCATTAACA-3′) to determine the expression level of the transgene. As for poplar metal transporters, specific forward and reverse primers were used to specifically amplify *PaMTP1* (5′-CTATCCACGAACTGCATATATG-3′ and 5′-CATTGTCTAGCACCATGTCTG-3′), *PaNRAMP1.3* (5′-GAAACAAGGAGGCTACACATC-3′ and 5′-TCCTCTGAGGCAACTGCATG-3′), *PaPCR2* (5′-TGGTTCATTGCTGCTGTGAGT-3′ and 5′-TACGCTGCGATTCTTCTTCTC-3′) and *PaHMA4* (5′-TCAGAGGTTCCTTTGATCGAG-3′ and 5′-CCTTAACAATTTGGAGCTGAGA-3′). Control genes for the analysis were selected as follows (forward and reverse primers are indicated): for *Arabidopsis*, the actin gene (5′-ATCCCAGTTGCTGACAATTC-3′ and 5′-GACCCGCCATACTGGTGTGAT-3′); for poplar, actin (5′-GAACTACGAGCTACCTGATG-3′ and 5′-CTTCCATTCCGATGAGCGAT-3′) and ubiquitin genes (5′-AAACAGCTTGAAGATGGAAGGA-3′ and 5′-TGTCTGAACTCTCCACCTCC-3′).

### Subcellular localization of ScZRC1 in *A. thaliana* protoplasts

To test the subcellular localization of ScZRC1 in plant, the expression vector pMD1-35S::*ScZRC1*-*eGFP* was produced by fusing the coding sequence of *ScZRC1* (amplified with the primers 5′-TCTAGAATGATCACCGGTAAAGAATTGA-3′ as forward and 5′-GTCGACCAGGCAATTGG AAGTATTGCA-3′ as reverse primers) in frame to the N-terminus of the reporter *eGFP* (amplified by PCR using 5′-GTCGACGTGAGCAAGGGCGAGGAGC-3′ as forward and 5′-GAGCTCTTACTTGTACAGCTCGTCCATG-3′ as reverse primers). As a control, a second expression vector pMD1-35S::*eGFP* was created by introducing the eGFP sequence (amplified by PCR using 5′-TCTAGAATGGTGAGCAAGGGCGAGGAGC-3′ as forward and 5′-GAGCTCTTACTTGTACAGCTCGTCCATG-3′ as reverse primers). *A. thaliana* protoplasts were then isolated and transfected with the two prepared constructs (pMD1-35S*::ScZRC1*-*eGFP* and pMD1-35S*::eGFP*) by PEG-mediated transfection, as previously described (Yoo et al. 2007). Images were taken with a Leica TCS SP5 confocal microscope using a 488 nm argon laser for the detection of GFP in the range of 496–555 nm, and both 488 nm and 561 nm lasers for the chloroplast auto-fluorescence detection at 651–800 nm.

### In-gel superoxide dismutase (SOD) activity assay and nitroblue tetrazolium (NBT) staining

Native soluble proteins were extracted from leaves harvested from poplar plants cultivated in hydroponic culture in control medium and supplemented with 500 µM ZnSO_4_ grinding fresh tissues in 0.15 M Tris, pH 7.5. Three independent poplar transgenic lines carrying 35S*::ScZRC1* or pRbcS*::ScZRC1* were analysed. After centrifugation, the supernatant was recovered and the total protein content was estimated using the DC protein assay (Bio-Rad Laboratories, Hercules, CA, USA). Proteins were separated at 4 °C by native 10% poly-acrylamide gel electrophoresis (PAGE) in Tris-glycine native buffer (25 mM Tris, 0.192 M glycine). SOD activity was then determined in-gel as described by Beauchamp and Fridovich (1971). The band intensity was determined by scanning the gels and processing images with Quantity OneR software v4.4.1 (Bio-Rad); intensities were normalized on actual protein loading, as estimated by Coomassie staining of a replica gel. O_2_^−^ in plants was detected by treating leaves with NBT and visualizing the blue spots according to Rao and Davis (1999).

### Quantification of metal content

Analysis of metal content was performed on plants grown in hydroponic culture and soil mesocosmos. Three independent transgenic lines carrying 35S*::ScZRC1* or pRbcS*::ScZRC1* for both species were analysed. Ten plants for wild type and each transgenic *A. thaliana* line were considered. Regarding poplar, four wild-type plants and each transgenic line were considered, either (i) grown in hydroponic culture on Hoagland’s solution treated with 500 μM ZnSO_4_ or 10 μM CdSO_4_ and 250 μM ZnSO_4_ or (ii) kept in soil artificially contaminated with 10 mg kg^−1^ CdSO_4_ and 300 mg kg^−1^ ZnSO_4_. The shoots and roots of *A. thaliana* plants and leaves, stems and roots of poplars were harvested. Samples were weighted and washed twice with ice-cold bidistilled water; shoots and roots were then oven dried at 60 °C for 72 h and ground to powder. Metal content was measured using inductively coupled plasma mass spectrometry (ICP-MS; EPA 6010C, 2007) after microwave-assisted acid digestion (EPA 3051A, 2007). Replica pots, filled with artificially contaminated soil (with 10 mg kg^−1^ CdSO_4_ and 300 mg kg^−1^ ZnSO_4_) were considered as non-planted control. At the end of the experiments, when plants were harvested, this control soils was air dried. Metal phytoavailability was determined by quantification by ICP-MS of Zn and Cd in solution upon CaCl_2_ extraction, which was demonstrated to provide the most useful indications of metal phytoavailability for studied elements (Qasim et al. 2005): 100 mL of 0.01 M CaCl_2_ were added to 10 g of soil, shaken for 2 h at 20 °C. The liquid to solid ratio of 10 was high enough to avoid sample heterogeneities (Qasim et al. 2005).

### Determination of chlorophylls

Fresh leaves were weighed, frozen and ground to a powder in liquid nitrogen, and chlorophylls were extracted with 80% acetone saturated with Na_2_CO_3_. Cell debris was removed by centrifugation at 10,000×*g*, 4 °C for 10 min, and the absorbance was determined at 750, 646.6 and 663.6 nm. The concentration of Chl *a* and *b* was determined as previously described (Porra 2002).

### Statistical analysis

Data in histograms are represented as mean ± standard deviation. Statistical significance of experimental data was evaluated using GraphPad Prism 7 (GraphPad Software). All analyses on *A. thaliana*, as well as chlorophyll content and expression analysis on poplar were subjected to a two-way analysis of variance (ANOVA) followed by a post hoc Bonferroni test. The remaining experiments on poplar were evaluated by means of a one-way ANOVA followed by a post hoc Tukey’s test. Statistically significant variations at *P* < 0.05 are marked with letters, the same letter corresponding to non-statistically significant differences.

## Results and discussion

The *ZRC1* gene encodes a transporter protein of the CDF family (Paulsen and Saier 1997). In *S. cerevisiae*, it transports Zn into the vacuole, reducing its concentration in the cytosol and protecting the cell from excess Zn (Miyabe et al. 2001; MacDiarmid et al. 2003). The overproduction of *ZRC1* conferred resistance to Zn excess, and the increased expression of *ZRC1* is a homeostasis mechanism in Zn stress tolerance (MacDiarmid et al. 2003). In the present study, *A. thaliana* was transformed with constructs carrying the *ScZRC1* coding sequence under the control of the CaMV35S promoter conferring constitutive expression, or under the control of the light-inducible promoter of the tobacco small subunit of Rubisco (pRbcS), to test the expression of *ScZRC1* only in the above-ground plant tissues. The promising results regarding Zn accumulation and tolerance in *A. thaliana* have brought us to investigate the effects of the expression of this transporter in the poplar clone Villafranca, a good candidate for metal phytoextraction due to its fast and consistent biomass production (Romeo et al. 2014).

### ScZRC1 localized to the vacuolar membrane in the plant cell

**Fig. 1:**
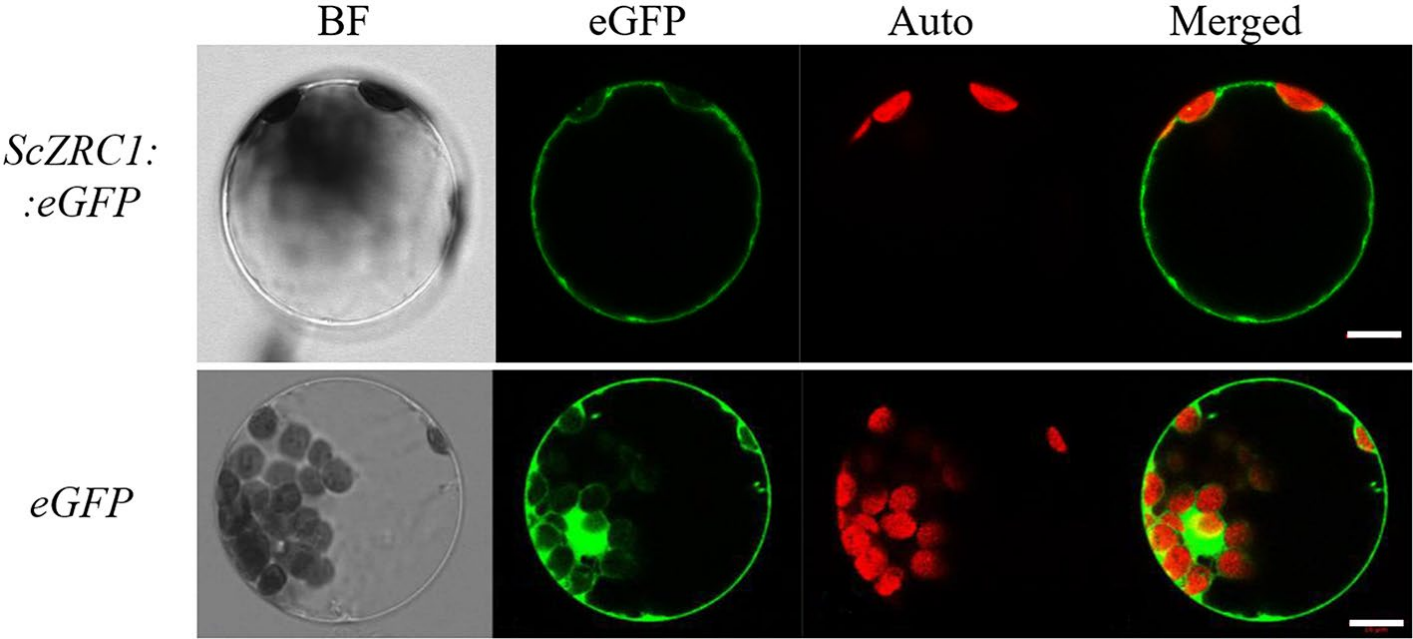
Subcellular localization of ScZRC1 in plant. *ScZRC1*::*eGFP* fusion protein (upper panel) and *eGFP* alone (cytoplasmic control, lower panel) were transiently expressed in *A. thaliana* leaf protoplasts by PEG-mediated transfection, and analyzed using fluorescence microscopy. BF, picture taken upon illumination in bright field; eGFP, fluorescence of the fusion protein *ScZRC1::eGFP*; Auto, chloroplasts revealed by chlorophyll autofluorescence; Merged, the two signals for eGFP and Auto were merged in the same image. Scale bar = 10 µm

To investigate the localization of the ScZRC1 protein in the plant cell, the construct carrying the *eGFP* sequence fused to the 3′-terminus of *ScZRC1* coding sequence was used for transient expression in *A. thaliana* protoplasts. Microscopic analysis of GFP fluorescence shows a cytosolic distribution of the eGFP alone, and a vacuolar membrane localization for the fusion protein ScZRC1::eGFP (Fig. 1), reflecting the tonoplast localization of the protein in yeast (Miyabe et al. 2001).

### ScZRC1 enhances Zn tolerance and accumulation in *A. thaliana*

Several *A. thaliana* p35S*::ScZRC1* and pRbcS*::ScZRC1* transgenic lines were obtained and the expression pattern of *ScZRC1* was assessed by real-time RT-PCR in roots and shoots of plants maintained in hydroponic culture. *ScZRC1* expression was confirmed in the whole plants for p35S*::ScZRC1* transgenic lines, whereas it was restricted to the shoots for plants harbouring the pRbcS*::ScZRC1* (Suppl. Fig. S1). Three independent transgenic lines for each construct were then chosen for further analyses.

**Fig. 2:**
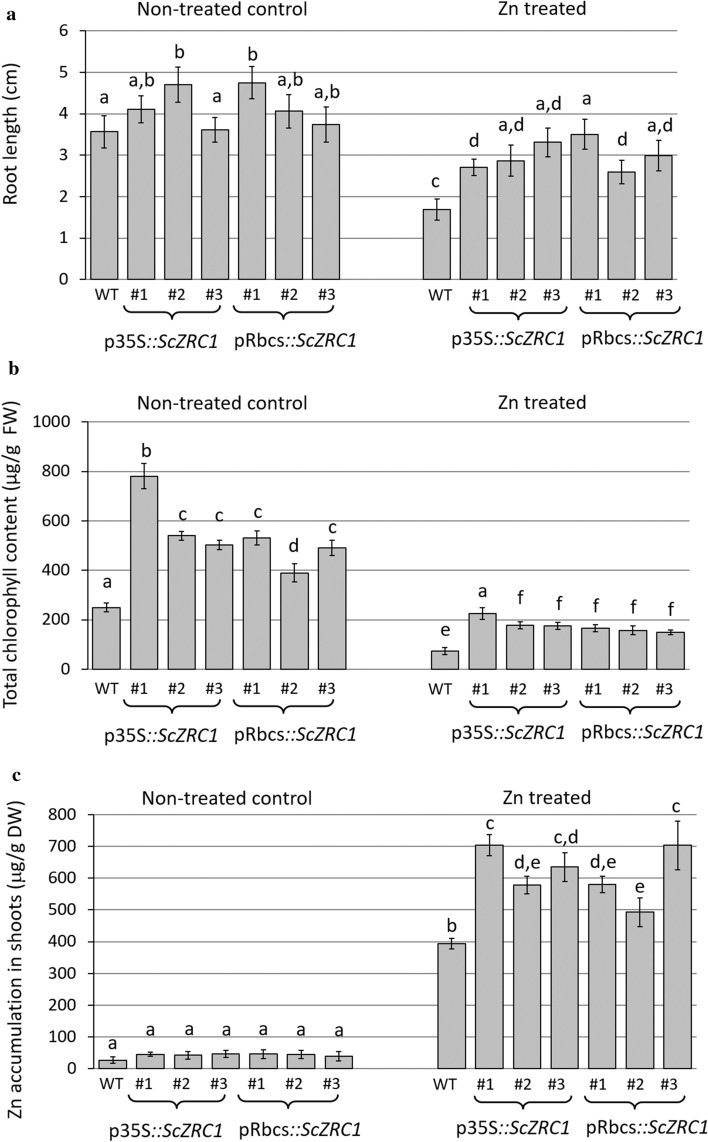
Analysis of Zn tolerance and accumulation in wild type (WT) and *ScZRC1*-expressing *A. thaliana* plants. Seeds were germinated on standard MS solid medium and grown in a growth chamber for 2 weeks. Plantlets were transferred in hydroponics for two further weeks, in standard Hoagland’s solution (non-treated control) and in Hoagland’s solution amended with 20 µM ZnSO_4_ (Zn treated). To estimate Zn tolerance, root length (**a**) and total chlorophyll content (**b**) were measured. Zn accumulation was determined in shoots (**c**). Different letters above the histograms indicate statistical significance, evaluated by two-way ANOVA followed by a post hoc Bonferroni test (*P* < 0.05, *n* = 30 for root length and *n* = 4 pools for chlorophyll content and Zn accumulation)

First of all, we evaluated the difference in Zn tolerance of transgenic lines by measuring root length and total chlorophyll content of plants after exposure to 0.7 μM ZnSO_4_ (control condition) and 20 μM ZnSO_4_ (excess Zn condition). Differences between wild type and transgenic lines were observed both in the absence and presence of Zn treatment (Fig. 2). In control conditions root length was not significantly different, with exception of one p35S*::ScZRC1* line, while upon treatment with excess Zn, root growth in all transgenic plants was significantly less inhibited than in wild-type plants (Fig. 2a). The chlorophyll content in all transgenic lines was significantly higher than in the wild type in both control and Zn treated conditions, even though the Zn treatment induced a marked and comparable decrease in chlorophylls in all genotypes (Fig. 2b). No differences were detected in either parameters between plants carrying p35S::*ScZRC1* or pRbcS::*ScZRC1* with the exception of line #1 p35S::*ScZRC1* and line #2 pRbcS::*ScZRC1*. The tested Zn tolerance indicators showed that *Arabidopsis* transgenic lines performed better than wild type even in control condition. In this respect, we speculate that the presence of the ScZRC1 transporter in the cellular tonoplast may contribute to improve/reinforce Zn acquisition and/or distribution. In addition, the effects of the transgene in Zn excess support the hypothesis that ScZRC1 confers enhanced tolerance to Zn excess. Overall, this transporter likely cooperates with other metal transporters, aiding metal homeostasis at least in the conditions tested.

Next, Zn accumulation was investigated in shoots of control plants and in *ScZRC1*-expressing transgenic lines. In control conditions, four out of six transgenic lines showed a significant increase of Zn accumulation with respect to wild-type plants. Under Zn excess, significantly more Zn accumulated in shoots of all but one of the *Arabidopsis ScZRC1*-expressing lines when compared to control plants (Fig. 2c). On the same samples Fe concentration was measured to verify whether the Zn excess could interfere and compete with Fe uptake and transport; no significant differences in Fe content were found in shoots between control plants and lines expressing *ScZRC1* (data not shown).

Altogether these results showed that ScZRC1 is localized to the tonoplast and hence its role in Zn tolerance may be exerted by vacuolar sequestration of the metal, analogously to its native role in yeast (Miyabe et al. 2001; MacDiarmid et al. 2003). As other members of the CDF family, such as MTPs in plants (van der Zaal et al. 1999; Arrivault et al. 2006), ScZRC1 enhances Zn accumulation both when constitutively expressed and when under the control of a light-inducible promoter. The significant accumulation of Zn in shoots of transgenic lines indicates that the production of ZRC1 in plants enhanced the long-distance Zn transport. Similar performances for Zn tolerance and accumulation were observed when the expression of *ScZRC1* was either under the control of a constitutive promoter or regulated by a light inducible promoter. Since the constitutive expression may adversely affect plant growth or determine abnormal development (Hsieh et al. 2002; Takasaki et al. 2010), the possibility of driving gene expression under the control of an inducible promoter received particular attention.

Highly efficient Zn accumulation in plants and storage capability allow to remove Zn from contaminated soils through a phytoremediation process. Considering this last aspect, we moved from *Arabidopsis* to *Populus*, analysing the effect of the expression of *ScZRC1* in transgenic poplar (a species which is worldwide recognized for its exploitation for phytoremediation, Dos Santos Utmazian et al. 2007). With this aim, we assessed Zn accumulation and tolerance in the presence of high Zn concentration in transgenic poplar lines harbouring p35S*::ScZRC1* and pRbcS*::ScZRC1*.

### ScZRC1 enhances Zn tolerance in *P. alba*

*ScZRC1* transcript abundance was analysed in different lines harbouring the p35S*::ScZRC1* or pRbcS*::ScZRC1* insert; for each construct, the three lines displaying the highest *ScZRC1* expression were selected for further trials. The different expression localization of *ScZRC1* determined by the 35S promoter or the light-inducible Rbcs promoter, tested by RT-PCR, is reported in Suppl. Fig. S1. All transgenic plants were similar to wild-type plants in appearance. To test whether *ScZRC1* expression in poplar can improve Zn tolerance and accumulation, plants propagated by cutting and maintained in vitro were moved to hydroponic culture on Hoagland’s solution for 2 weeks, and then treated with 500 µM ZnSO_4_ for further 3 weeks before proceeding to the phenotypic assays.

**Fig. 3:**
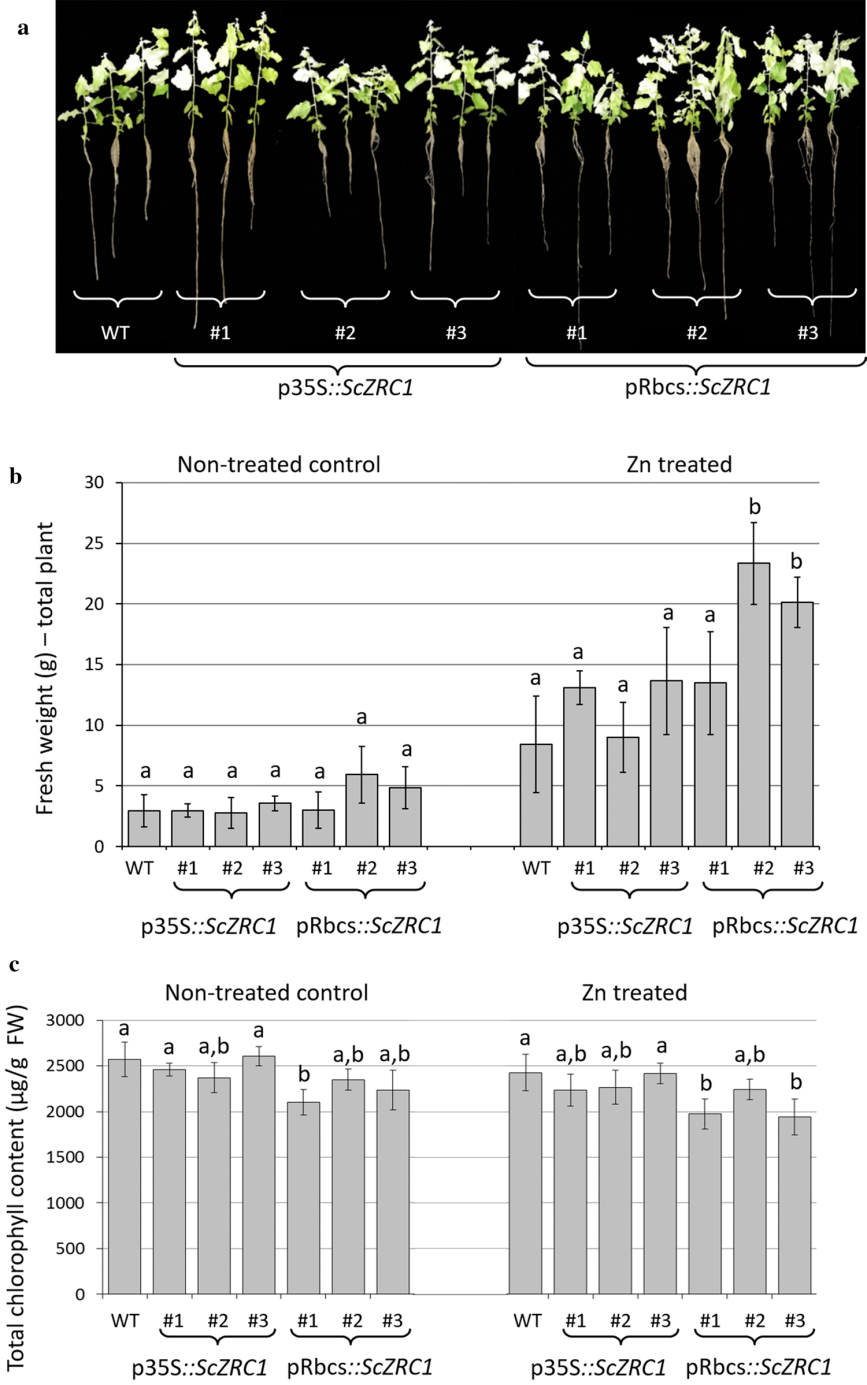
Zn tolerance of poplar plants, wild type (WT) and transgenic harboring p35S*::ScZRC1* and pRbcs*::ScZRC1*, grown hydroponically for 2 weeks in Hoagland’s solution and for three further weeks in Hoagland’s solutions supplemented with 500 μM ZnSO_4_. Plants were evaluated before and after Zn treatment. General phenotype **a** total fresh weight of the plant **b** and total chlorophyll content **c** were measured to determine Zn tolerance. Different letters above the histograms indicate statistical significance, evaluated by one-way ANOVA followed by a post hoc Tukey’s test (*P* < 0.05, *n* = 5) for fresh weight analysis and by two-way ANOVA followed by a post hoc Bonferroni test (*P* < 0.05, *n* = 5) for chlorophyll content

Transgenic plants obtained after transformation with both constructs did not show distinguishable differences in visual appearance with wild-type plants after the treatment with 500 µM ZnSO_4_ (Fig. 3a). Due to the inaccuracy in measuring root length in micropropagated poplar plants, we decided to consider the plant fresh weight and chlorophyll content as indicators for tolerance. Fresh weight was measured on the same plants before and after the Zn treatment. No differences were observed between control and *ScZRC1*-expressing lines or between plants carrying the p35S::*ScZRC1* and pRbcS::*ScZRC1* cassettes for lines #2 and #3 of pRbcs::*ScZRC1* which appear to be taller before the treatment and upon Zn treatment (Fig. 3b). In addition, regarding chlorophyll content, similar results were found: the total amount of chlorophyll was unchanged before and after the Zn treatment, with the exception of lines 1 and 3 of pRbcs*::ScZRC1*, which show a small decrease in chlorophyll concentration when compared to wild type control, but no changes upon Zn treatment (Fig. 3c).

**Fig. 4:**
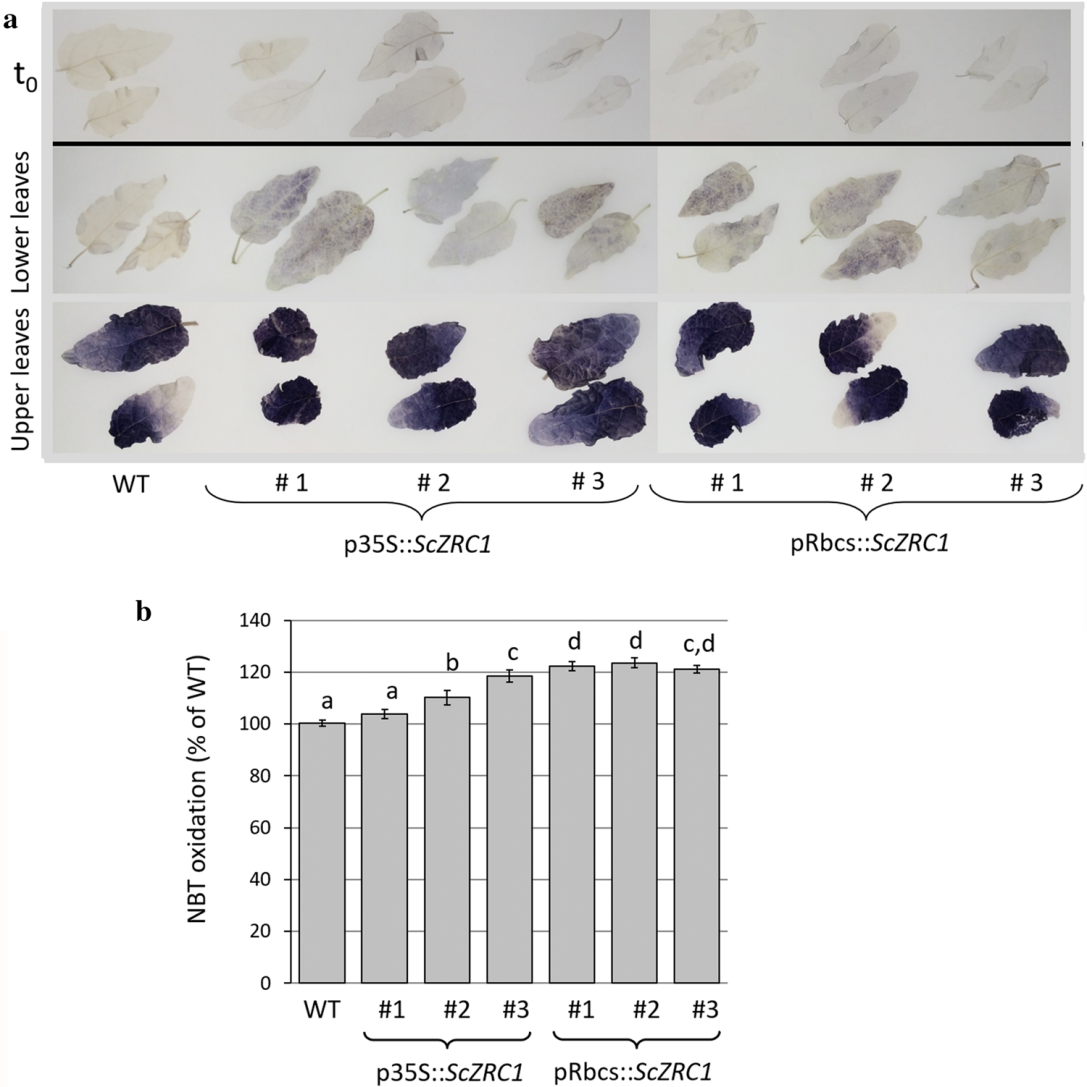
Oxidative stress in poplar plants, wild type (WT) and transgenic harboring p35S*::ScZRC1* and pRbcs*::ScZRC1*, grown hydroponically for 2 weeks in Hoagland’s solution and for three further weeks in Hoagland’s solutions supplemented with 500 μM ZnSO_4_. **a** Nitroblue tetrazolium (NBT) analysis on leaves of plants before (t0, upper panel) and after Zn treatment (two lower panels). Zn-treated lower leaves (shaded by the other leaves) and upper leaves (in full light) were collected and analyzed. **b** In-gel SOD activity, measured on total protein extracts from leaves of Zn-treated plants. SOD activity is expressed as a percentage of NBT oxidation in comparison to the WT value. Results are derived from densitometric analysis of visibly clear bands in an in-gel activity assay. Each measurement was performed in triplicate. Band intensity was normalized on the actual protein loading. Different letters indicate significantly different values, evaluated by one-way ANOVA followed by a post hoc Tukey’s test (*P* < 0.05, *n* = 5)

Further analyses were performed to determine the potential increase in reactive oxygen species (ROS) in leaves by nitroblue tetrazolium (NBT) staining which detects the superoxide anion radical (${\text{O}}_{2}^{ - \cdot }$). In all plants maintained in hydroponic culture and treated for 3 weeks with 500 µM ZnSO_4_, an evident increase in oxidative stress was observed on upper leaves as effect of the light intensity in the growth chamber, whereas a pale colouring indicating a slight superoxide anion accumulation was seen in the lower leaves of all plants expressing *ScZRC1* (Fig. 4a). In addition, the sensitivity to ROS on the same plants was measured by detecting in-gel SOD activity, on total protein extracts from leaves. This analysis showed a slight but significant increase of SOD activity in all *ScZRC1*-expressing lines but one (Fig. 4b). These data indicate that the high Zn accumulation in shoots of transgenic lines stimulates a moderate production of free radicals, imposing oxidative stress and leading to the activation of antioxidant defence mechanisms that help contain the cellular damage, as reported also in other species (Małecka et al. 2019). Therefore, *ScZRC1*-expressing plants might have acquired a more efficient antioxidant system with increased activity of antioxidant enzymes, which in turn is associated with tolerance to high Zn concentrations.

### ScZRC1 enhances Zn accumulation in *P. alba*

**Fig. 5:**
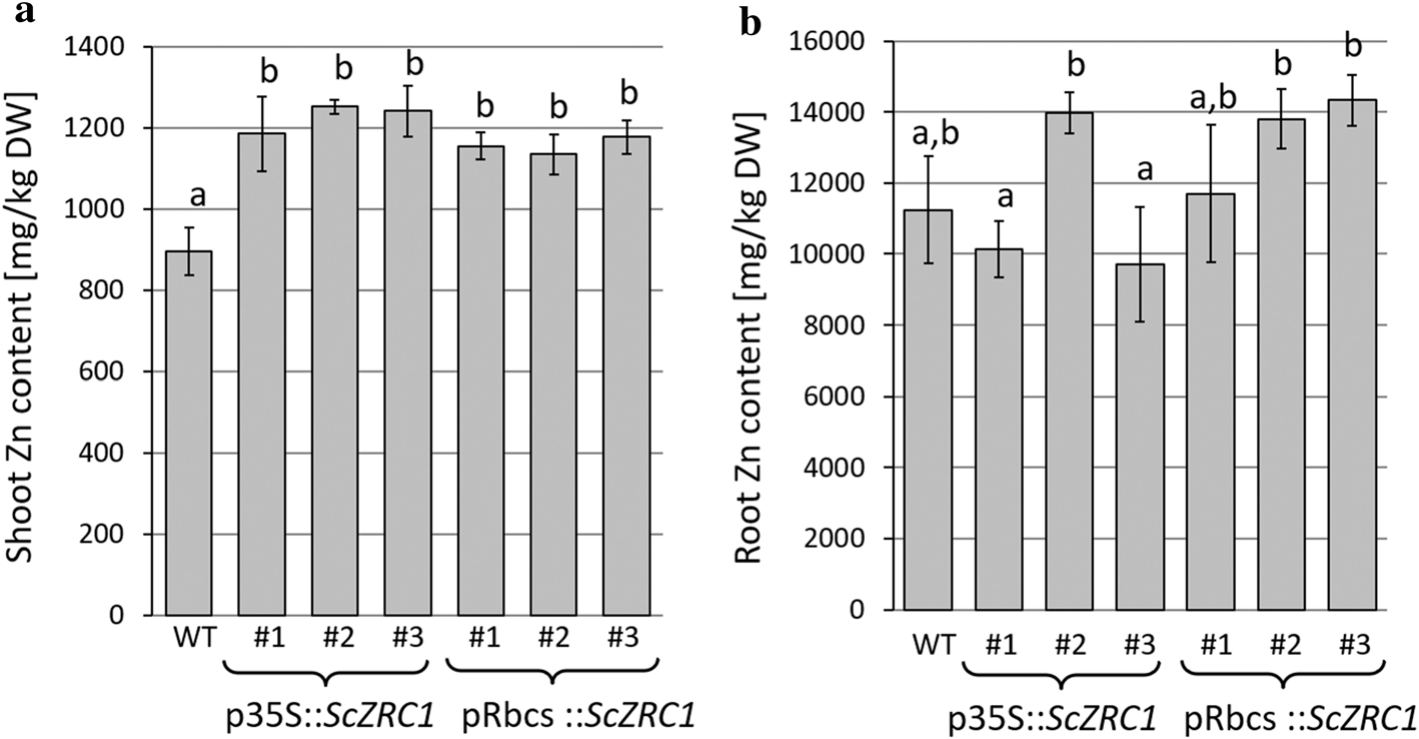
Analysis of Zn content in shoots **a** and roots **b** of poplar plants, wild type (WT) and transgenic harboring p35S*::ScZRC1* and pRbcs*::ScZRC1*, measured after 3 weeks of growth in Hoagland’s solution upon addition of 500 µM ZnSO_4_. Different letters above the histograms indicate statistical significance, evaluated by one-way ANOVA followed by a post hoc Tukey’s test (*P* < 0.05, *n* = 5)

**Fig. 6:**
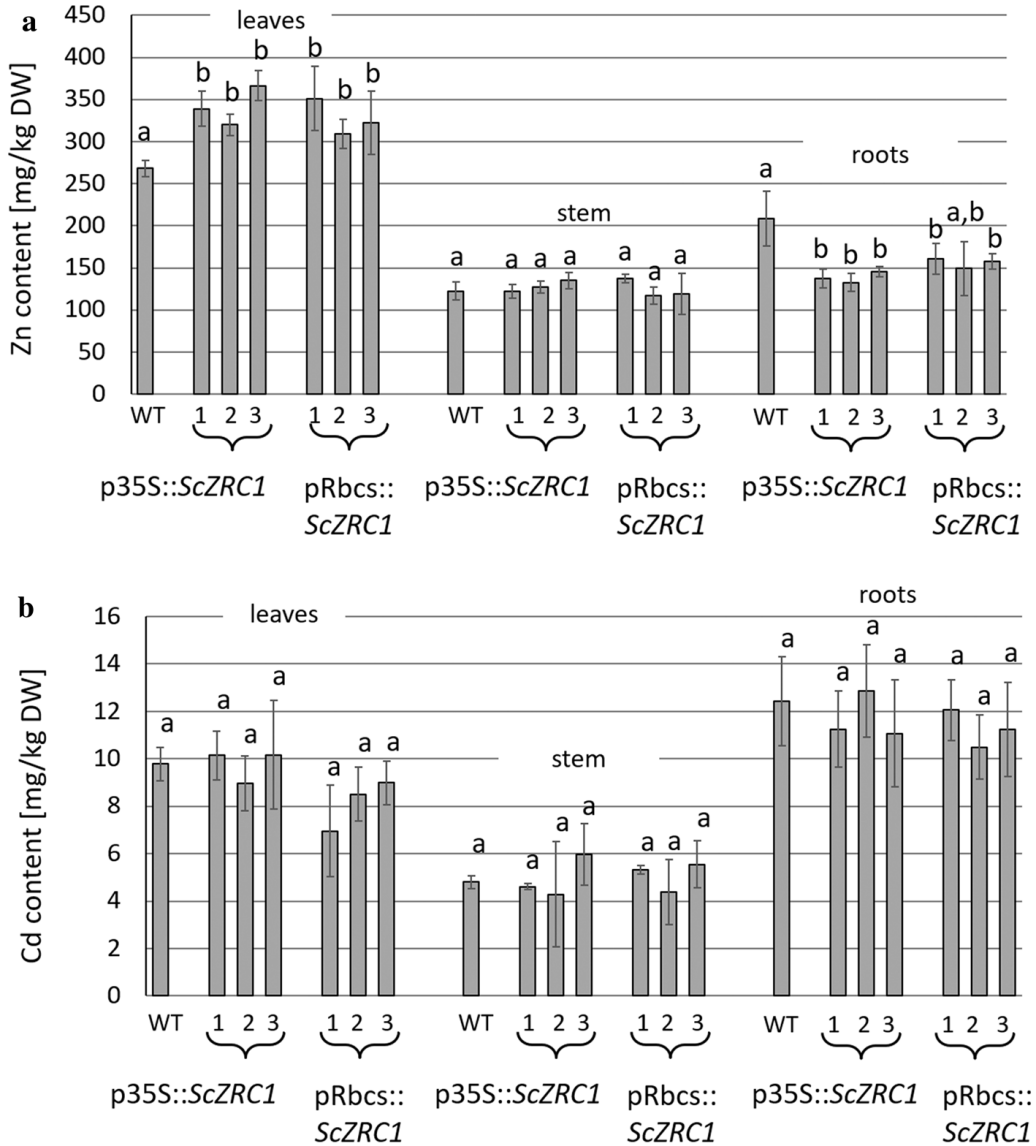
Analysis of Zn (**a**) and Cd (**b**) in leaves, stems and roots of poplar plants, WT and *ScZRC1*-expressing plants. Metals were analyzed after 3 weeks of growth in mesocosmos pots filled with soil artificially contaminated with 10 mg kg^−1^ CdSO_4_ and 300 mg kg^−1^ ZnSO_4_. Different letters above the histograms indicate statistical significance, evaluated by one-way ANOVA followed by a post hoc Tukey’s test (*P* < 0.05, *n* = 5)

Zn content was analysed in the shoots and roots of plants grown in hydroponic solution. All transgenic poplar lines, regardless of which promoter the *ScZRC1* expression was driven by, accumulated significantly higher levels of Zn in their shoot than the untransformed control (Fig. 5a), whereas no clear differences were found in Zn concentration in roots between control plants and *ScZRC1*-expressing lines (Fig. 5b). It is worth noting that the poplar clone Villafranca, chosen for this study, was the least damaged and showed the highest Zn tolerance and accumulation capacity, among four commercial clones, when grown in modified Hoagland's nutrient solution under Zn excess for 4 weeks (Romeo et al. 2014). Thus, the absence of toxicity symptoms after exposure to 500 µM ZnSO_4_ for 3 weeks, observed even in wild-type plants matches the intrinsic tolerance of the Villafranca clone to high Zn concentrations. The poplar lines expressing *ScZRC1* were able to take up, transport from root to shoot and accumulate more Zn in the aerial part than the wild type and possess the characteristics that increase the potential of poplar for phytoextraction of Zn from polluted soils. Such enhanced Zn accumulation in shoots of transgenic individuals prompted us to test the potential accumulation capacity of these plants in the presence of multimetallic mixtures, as often happens in polluted soils. Therefore, poplar plants were tested both in mesocosmos pots filled with an artificially contaminated soil substrate (10 mg kg^−1^ CdSO_4_ and 300 mg kg^−1^ ZnSO_4_) and in hydroponic culture (10 μM CdSO_4_ and 250 μM ZnSO_4_), considering that these metals are frequently co-pollutants and share several chemical properties (Chaney 2010). Even in the presence of both, Zn and Cd plants did not show visible symptoms of metal toxicity, further supporting the intrinsic metal tolerance of this poplar clone (data not shown). After 3 weeks of growth in the contaminated soil substrate, poplar plants harbouring the *ScZRC1* gene accumulated higher amounts of Zn in the shoot, particularly in leaf tissues, while Zn accumulation in roots decreased in transgenic individuals when compared to wild-type controls (Fig. 6a). The Zn translocation factor, calculated as the ratio between metal concentration in shoots and metal concentrations in roots, is increased in plants harboring *ScZCR1* (Table 1). These evidences point to an enhanced effectiveness of the root-to-shoot transport of Zn due to the expression of this vacuolar yeast Zn transporter. The enhanced Zn accumulation capacity is also demonstrated by the increased total Zn accumulated by the plant tissues, as reported in Table 2.

**Table 1:** Zn and Cd translocation factors

Genotype	Zn translocation factor		Cd translocation factor	
WT	1.31a	± 0.25	0.83a	± 0.18
p35S::*ScZRC1* #1	2.47b	± 0.05	0.7a,b	± 0.02
p35S::*ScZRC1* #2	2.43b	± 0.29	0.59a	± 0.44
p35S::*ScZRC1* #3	2.52b	± 0.23	0.91a	± 0.54
pRbcs::*ScZRC1* #1	2.18c	± 0.03	0.57a,b	± 0.10
pRbcs::*ScZRC1* #2	2.13b,c	± 0.57	0.95a	± 0.40
pRbcs::*ScZRC1* #3	2.04c	± 0.02	0.85a	± 0.34

Zn and Cd translocation factors (means ± SD) were calculated as the ratio between metal concentration in shoots and metal concentrations in roots of poplar plants (three lines each transgenic genotpye indicated as #1, #2 and #3) grown in mesocosmos pots filled with an artificially contaminated soil substrate (10 mg kg^−1^ CdSO_4_ and 300 mg kg^−1^ ZnSO_4_). The translocation factor is a measure for the effectiveness of the root-to-shoot transport of a metal ion. Different letters (a, b, c) within a column indicate significant differences among the genotypes (*P* < 0.05, *n* = 5)

**Table 2:** Total metals in harvestable parts

Genotype	plant dry weight (g)		Zn content (µg)		Cd content (µg)	
WT	0.87	± 0.21	185.66a	± 6.66	6.91a	± 0.62
p35S::*ScZRC1* #1	0.96	± 0.21	248.56b	± 59.13	7.07a	± 3.11
p35S::*ScZRC1* #2	1.02	± 0.25	244.3b	± 48.13	6.16a	± 2.92
p35S::*ScZRC1* #3	0.93	± 0.13	247.51b	± 33.45	7.51a	± 3.56
pRbcs::*ScZRC1* #1	0.87	± 0.17	239.15b	± 26.42	5.66a	± 2.07
pRbcs::*ScZRC1* #2	1.05	± 0.05	246.87b	± 12.84	7.29a	± 0.12
pRbcs::*ScZRC1* #3	1.08	± 0.28	254.13b	± 57.96	7.97a	± 0.89

Poplar biomass and Zn and Cd content in total harvestable tissues, leaves plus stems (means ± SD). The numbers #1, #2 and #3 indicate three different lines used. Different letters (a and b) within a column indicate significant differences among the genotypes (*P* < 0.05, *n* = 5)

Differently, the expression of *ScZRC1*, either constitutive or circumscribed to the green tissue, did not result in an altered accumulation of Cd (Fig. 6b). The translocation factor associated to this metal was unchanged between wild type and transgenic plants (Table 1). In the experiment conducted in hydroponic culture, with double contamination of 10 μM CdSO_4_ and 250 μM ZnSO_4_, no differences were observed in Zn shoot accumulation between control and transgenic lines (Suppl. Fig. S2), whereas the amount of Zn in roots was significantly higher in wild-type plants than in *ScZRC1*-expressing plants. Likewise, measurement of Cd concentration in these plants revealed similar values of Cd content in shoots of control and transgenic lines, while a significantly lower Cd accumulation was measured in roots of five out of six transgenic lines compared to wild-type control (Suppl. Fig. S2).

Interestingly, the full potential of poplar plants in accumulating Zn was not reached in soil conditions. Even if the concentration of total Zn in soil is much higher than the concentration in hydroponic solution, the bioavailability of this metal in soil is greatly decreased by the interaction between the metal ions and the soil matrix itself (Rooney et al. 2006). Bioavailable metals in the pot mesocosmos were estimated as 3.30 ± 0.24 mg kg^−1^ for Zn and 0.10 ± 0.05 mg kg^−1^ for Cd. Such bioavailability, significantly lower than that of the liquid nutrient solution, reflects in the difference in accumulated metals between hydroponic conditions and soil. Metal concentrations in shoots of plants grown in hydroponic culture are up to four times higher for Zn and more than 20 times higher for Cd than in soil, and the difference is even greater for roots (Fig. 6 and Suppl. Fig. S2). The data obtained in hydroponics, apparently contrasting with the results achieved in soil, suggest a competition between Zn and Cd, at least when plants are cultured in hydroponic solution, and the availability of the two metals is greatly higher than in soil. There are evidences that Zn and Cd uptake and distribution in plants can be at least partially competitive (Noraho and Gaur 1995; Queiroz Santos et al. 2014; Fontanili et al. 2016). Moreover, it has been highlighted in previous works that root-to-shoot translocation (here estimated as translocation factor) is strongly dependent on the growth conditions and the metal availability and generally inversely proportional to the latter (Talke et al. 2006; Fernandez et al. 2012; van der Ent et al. 2013; Kozhevnikova et al. 2017), consistently with what was observed in this analysis. It must also be considered that strategies based on hydroponic culture with high metal concentrations can lead to the saturation of the plant system, thus flattening the differences in metal accumulation (van der Ent et al. 2013). Despite these flaws of hydroponics, both the strategies tested support a greater effectiveness of *ScZRC1*-expressing plants in Zn translocation from roots to shoots, which becomes manifest in a higher Zn accumulation in shoots in the first analysis and a lower Zn and Cd retention in roots in the second. Therefore, the potential of *ScZRC1*-expressing poplar plants for phytoremediation is evident, but their efficiency has to be assessed case-to-case in field soils contaminated with multiple heavy metals. Under in-field conditions, characterized by a multi-year timeframe, the biomass developed by poplar plants, the deeper root apparatus and the interactions of roots with rhizosphere microorganisms will influence the remediation capacity, allowing to drive conclusions on the applicability in field of these transgenic clones.

### Influence of ScZRC1 on the expression of metal transporters in *Populus alba*

**Fig. 7:**
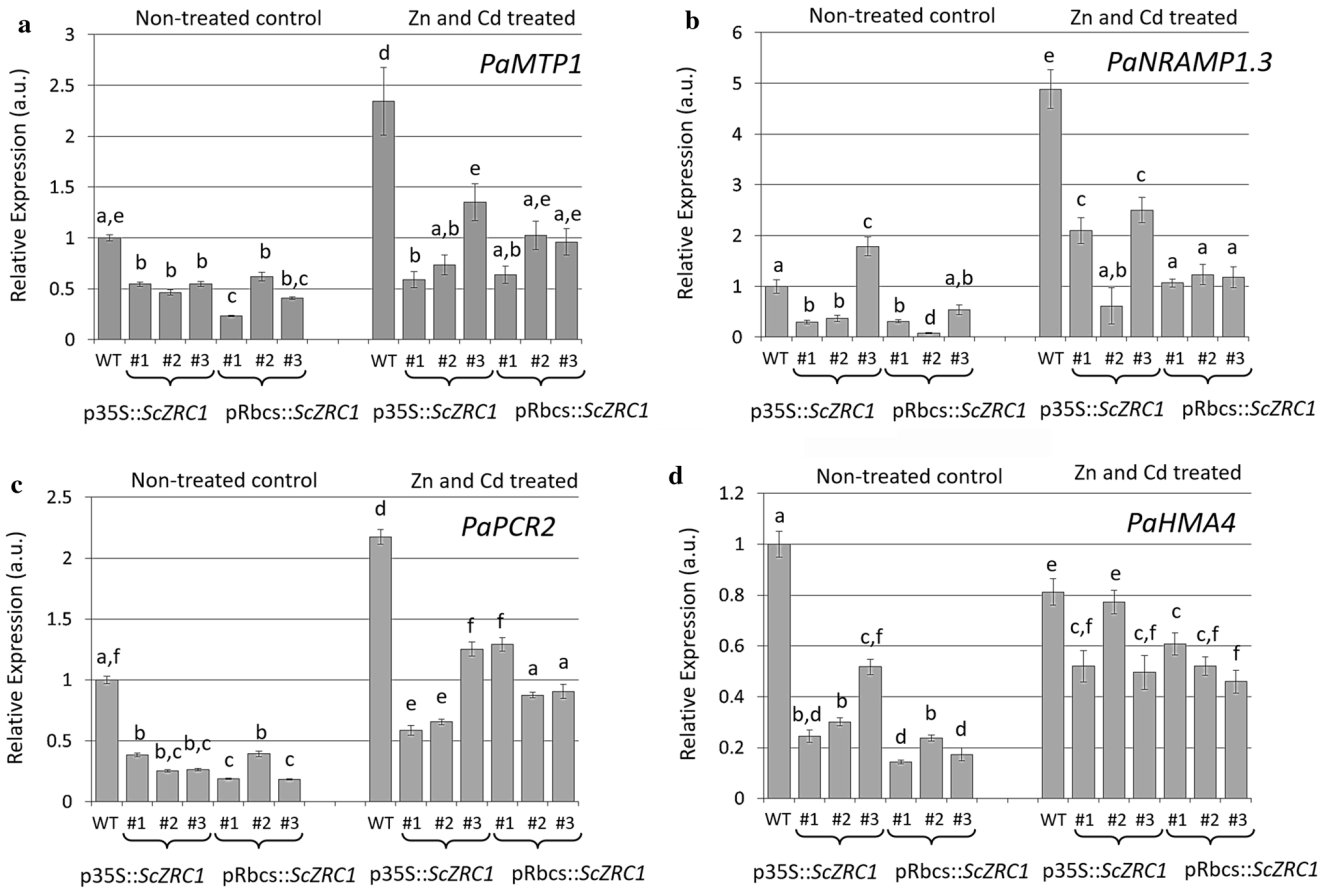
Real-time RT-PCR analysis on the expression of poplar transporters *PaMTP1* (**a**), *PaNRAMP1.3* (**b**), *PaPCR2* (**c**) and *PaHMA4* (**d**) in leaves of wild type and *ScZRC1*-expressing plants, exposed to high Zn and Cd in comparison to control conditions. The expression levels were calculated using the 2^−ΔΔCT^ method, relative to the expression level in leaves from WT plants upon untreated conditions. Different letters indicate statistical significance, evaluated by two-way ANOVA followed by a post hoc Bonferroni test (*P* < 0.05, *n* = 3)

As described in the previous paragraph, the results achieved for metal accumulation in poplar suggest a competition between Zn and Cd or possibly other ions in the nutrient medium, and a possible interference in root-to-shoot transport. To understand if metal homeostasis as a whole is altered by the introduction of *ScZRC1*, we monitored the expression modulation of the genes encoding four poplar metal transporters in *ScZRC1* transgenic plants grown in hydroponic condition with both Zn and Cd present in the medium (Fig. 7). For this analysis we selected poplar homologues of the following genes: (i) the metal tolerance protein 1 (*MTP1*), localized in the tonoplast and involved in Zn tolerance by metal sequestration into the vacuole (Gustin et al. 2009; Podar et al. 2012); (ii) the natural resistance-associated macrophage protein 1.3 (*NRAMP1.3*), whose homologous AtNRAMP1 of *Arabidopsis* is located in the plasma membrane and controls entry of different elements in the cytosol (Cailliatte et al. 2010; Castaings et al. 2016); (iii) the plant cadmium resistance protein 2 (*PCR2*) and (iv) the P-type heavy metal ATPase 4 (*HMA4*) which are also located in the plasma membrane, where they translocate cytosolic bivalent cations (e.g., Cd^2+^ / Zn^2+^) for xylem loading (Hanikenne et al. 2008; Song et al. 2010). These genes are modulated in response to Cd treatment in poplar lines overexpressing γ-glutamylcysteine synthetase (He et al. 2015a, b). As reported in Fig. 7, increased transcription for *PaMTP1*, *PaNRAMP1.3* and *PaPCR2* was detected in leaves of wild-type plants when exposed to high Zn and Cd, in comparison to control conditions. The expression of *ScZRC1* in transgenic lines induced a reduction in the expression of all the four considered transporters also in control conditions, with a slight induction upon metal treatment. With reference to existing literature, the analysis of metal transporters highlights a variable behaviour, highly dependent on the species analysed, the organ examined and the treatment applied. For instance, the expression of *MTP1* decreases in roots of *Populus tremula* × *Populus alba* upon Cd treatment for 80 days (He et al. 2015a, b), while it is enhanced by Zn or Cd treatment in roots of the metal hyperaccumulator *Noccaea caerulescens* (Ganges ecotype) (Martos et al. 2016). In the present experiment, *PaMTP1* upregulation in wild-type plants under metal treatment is consistent with its role in Zn detoxification. Moreover, the significantly lower levels measured in *ScZRC1*-expressing plants in both control and treated conditions can be easily explained by the likely overlapping roles of ScZRC1 and PaMTP1 in metal vacuolar storage (Fig. 7a). As for *PaNRAMP1.3*, variable results have been reported in previous studies. For example, *NRAMP1.3* upregulation in roots was induced by ABA treatment in *Populus *×* canescens,* whereas its level in shoots decreased upon Pb exposure regardless of exogenous ABA addition (Shi et al. 2019). Moreover, *NRAMP1.3* upregulation in roots was detected in poplar plants overexpressing the γ-glutamylcysteine synthetase, and upon Cd treatment such induction was enhanced more in transgenic individuals than in wild-type plants (He et al. 2015a, b). In *A. thaliana,* PCR2 plays a role in Zn detoxification and root-to-shoot transport thus conferring tolerance to excess Zn and Cd (Song et al. 2010), while in *Populus* × *canescens*, its transcription was induced by Zn excess in roots but not in shoots (Shi et al. 2015).

Conversely to the other genes tested, mRNA level of *PaHMA4* did not change in leaves of wild-type plants exposed to Zn and Cd excess. On the other hand, a slight increase was observed in all *ScZRC1*-expressing lines upon Zn and Cd treatment compared to control condition (Fig. 7d). In *A. thaliana*, HMA4 plays an essential role in root-to-shoot translocation of Zn and Cd, to maintain Zn homeostasis and Cd detoxification (Hussein et al. 2004). In poplar cells, this transporter is implicated in pumping Zn^2+^ out of the cytosol; in this species, a down-regulation of *HMA4* mRNA levels was reported in response to excess Zn (Adams et al. 2011).

Overall, this transcriptional analysis suggests an alteration in the global metal homeostasis, as highlighted by the lower mRNA levels of the four transporter tested in plants expressing *ScZRC1* than in wild type. Therefore, it may be hypothesised that ScZRC1 collaborates with other transporters in the management of Zn and Cd excess; in particular, due to the similarity in localization and activity, a functional overlapping can be suggested with PaMTP1.

## Conclusions

In summary, ScZRC1 is a vacuolar transporter able to confer Zn tolerance and accumulation in shoot when expressed in plants. The increased Zn concentration in the shoot was observed in particular in poplar, a species suitable for phytoremediation. In a comparison with other clones, the Villafranca used in this study performed better in terms of Zn tolerance and accumulation (Romeo et al. 2014). The experiments conducted in the present study suggest that it is possible to even increase the potential of this clone for the remediation of Zn polluted soils. However, the results achieved when Cd was present in addition to Zn indicate that case-to-case field trials, over a multi-year timeframe allowing substantial plant growth, are required when dealing with multimetal polluted soils.

### *Author contribution statement*

GDC, FM: conception and design of the study, acquisition of data, analysis and interpretation of data; GDC, AM, EF, GV and AF: drafting the article and revising it critically for important intellectual content; AF: Funding acquisition; GDC, FM, AM, EF, GV and AF final approval of the version to be submitted.

## Supplementary Information

Below is the link to the electronic supplementary material.

Supplementary Fig. S1 Expression of ScZRC1 in transgenic *Arabidopsis* and poplar lines. Fig. S2 Quantification of Zn and Cd in shoots and roots of poplar plants grown in hydroponic conditions (DOCX 669 KB)

## Abbreviations

eGFP: Enhanced green fluorescent protein
HM: Heavy metal ATPase
MTP1: Metal tolerance protein 1
NRAMP: Natural resistance-associated macrophage protein
PCR2: Cadmium resistance protein 2
RbcS: Rubisco small subunit
SOD: Superoxide dismutase
ZRC1: Zinc/cadmium resistance protein
