# Supplementary material for: Enhancement of Zn tolerance and accumulation in plants mediated by the expression of Saccharomyces cerevisiae vacuolar transporter ZRC1
Source: Planta. 2021 May 6;253(6):117. doi: 10.1007/s00425-021-03634-z (PMC8102461; doi:10.1007/s00425-021-03634-z)
Supplement: Supplementary file 1 — Supplementary Fig. S1 Expression of ScZRC1 in transgenic Arabidopsis and poplar lines. Fig. S2 Quantification of Zn and Cd in shoots and roots of poplar plants grown in hydroponic conditions (DOCX 669 KB) [file 425_2021_3634_MOESM1_ESM.docx]

**Enhancement of Zn tolerance and accumulation in plants mediated by the expression of *Saccharomyces cerevisiae* vacuolar transporter *ZRC1***

Giovanni DalCorso^1^*, Flavio Martini^1^*, Elisa Fasani^1^, Anna Manara^1^, Giovanna Visioli^2^ and Antonella Furini^1^**^†^**

**SUPPLEMENTARY MATERIAL**

**a - *Arabidopsis***


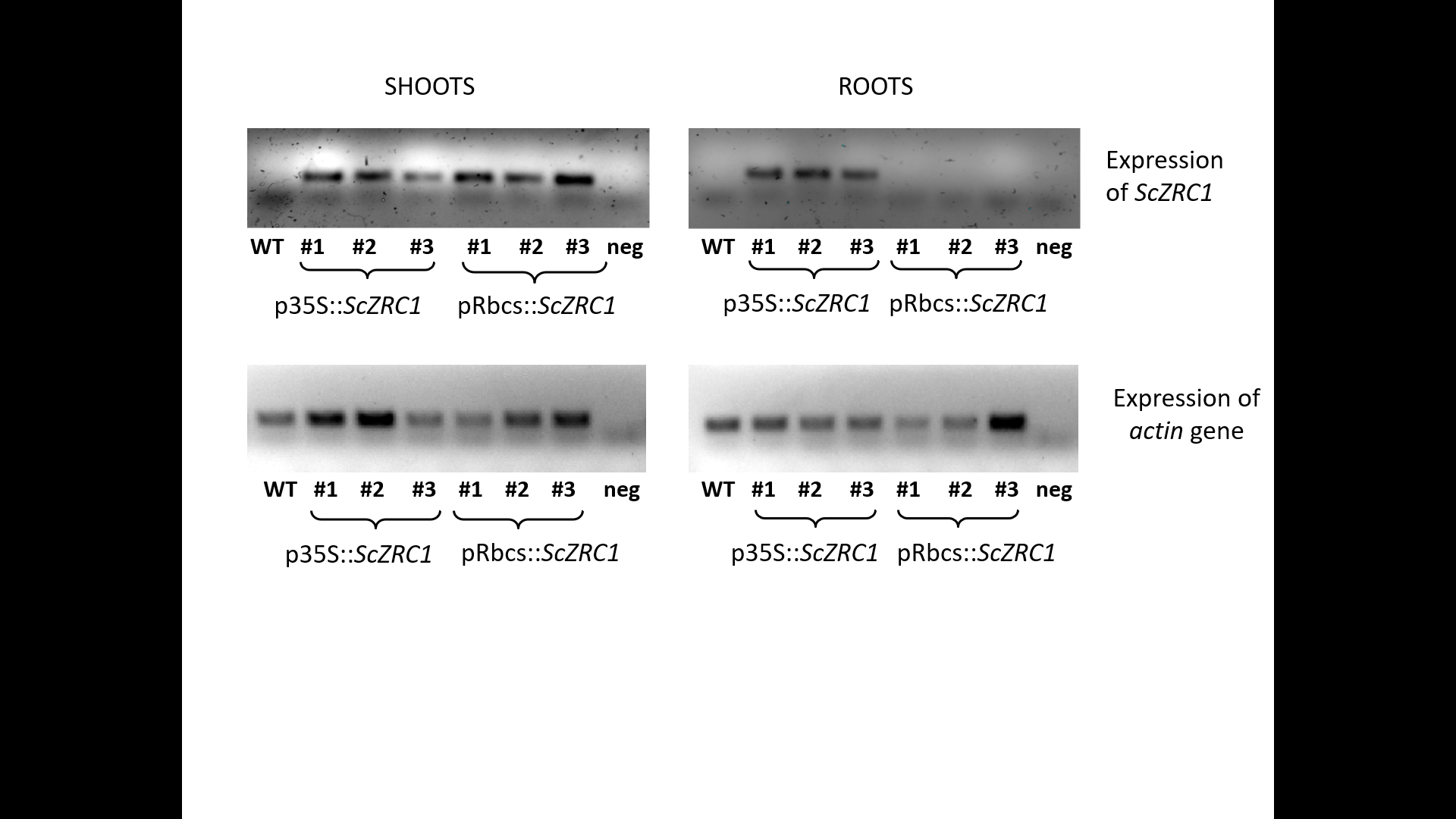


**b - Poplar**


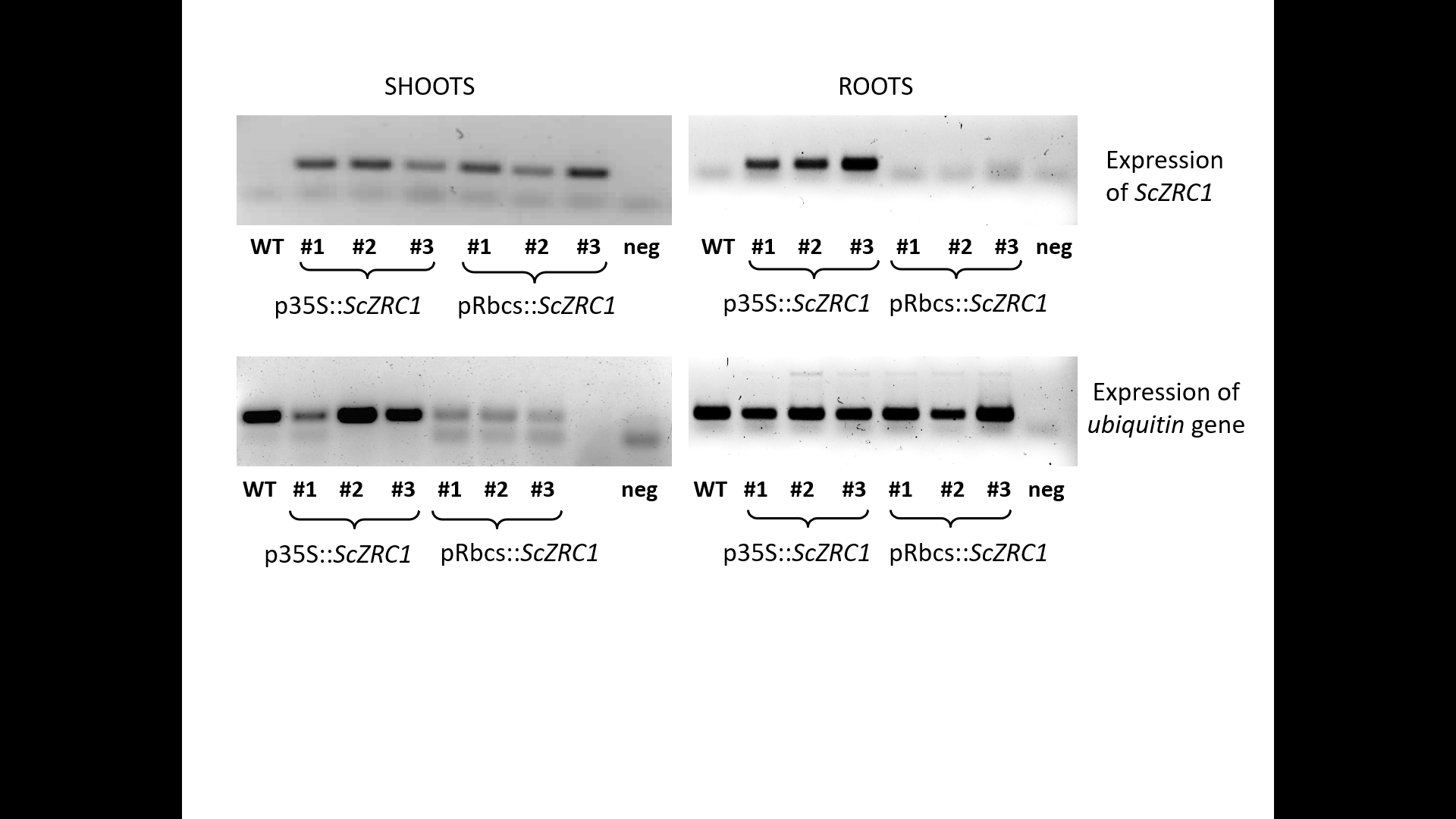


**Supplementary Figure S1**: Expression of *ScZRC1* in transgenic *Arabidopsis* and poplar lines. Transcripts were detected by RT-PCR. PCR products obtained after 40 cycles with *ScZRC1*-specific primers and control primers for the actin (panel **a**, in *Arabidopsis*) or *ubiquitin* gene (panel **b,** in poplar) were analysed on agarose gels. “**neg**” indicates the negative control, in which water has been used instead of cDNA template. (see Material and Methods for detailed description of RNA purification, cDNA synthesis and primer sequences).


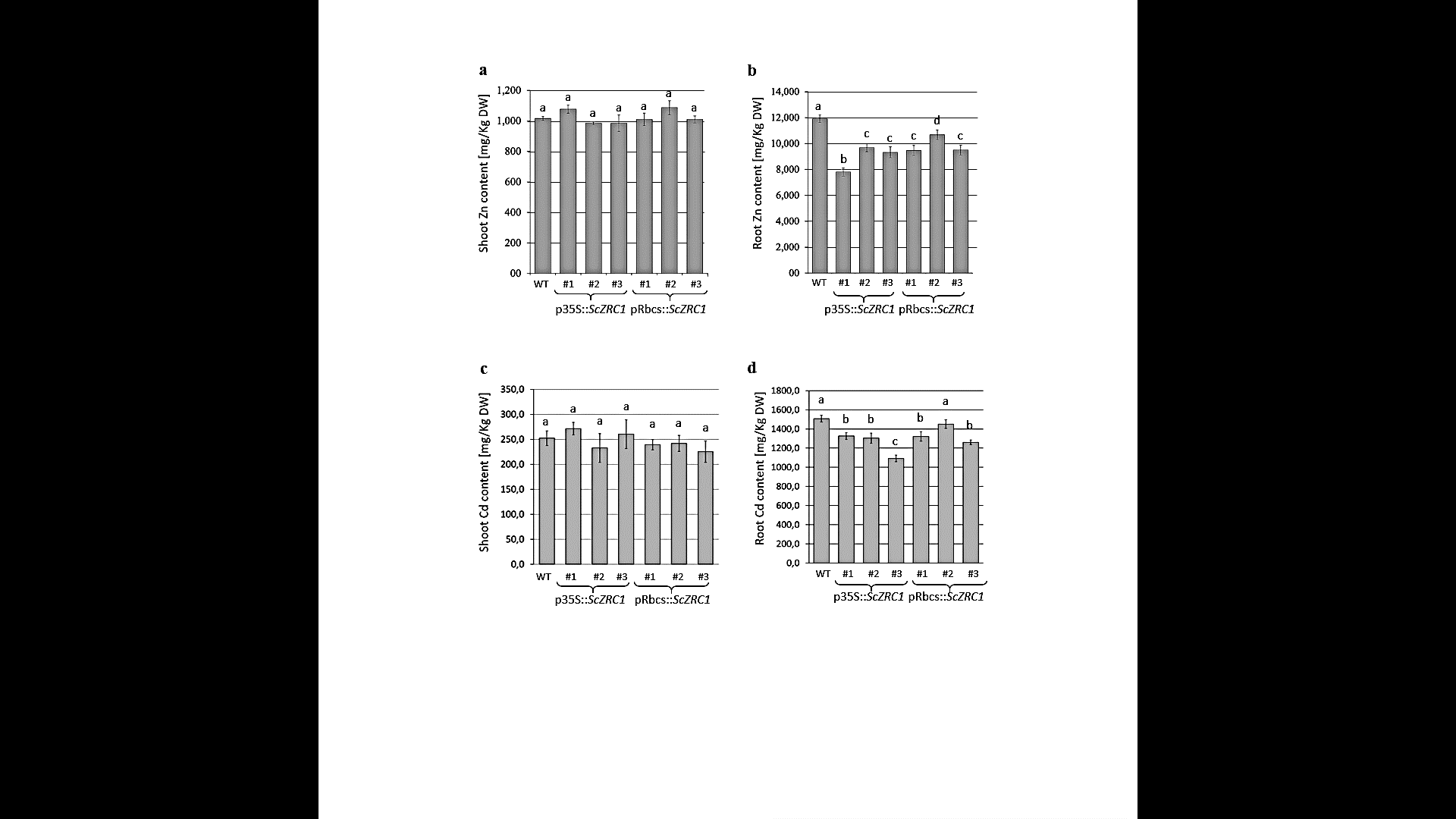


**Supplementary Figure S2**: Analysis of Zn (**a** and **b**) and Cd (**c** and **d**) in shoots and roots of poplar plants, WT and ZRC1 expressing plants. Metals were analysed after three weeks in Hoagland’s solution amended with 10 µM CdSO_4_ and 250 µM ZnSO_4_. Different letters above the histograms indicate statistical significance, evaluated by one-way ANOVA followed by a post hoc Tukey’ test (*P* < 0.05, *n* = 5).
